# Influence of Transcranial Direct Current Stimulation and Exercise on Physical Capacity and Gait in Multiple Sclerosis: A Cross-Over Pilot Study

**DOI:** 10.3390/healthcare11101384

**Published:** 2023-05-11

**Authors:** Inés Muñoz-Paredes, Azael J. Herrero, Jesús Seco-Calvo

**Affiliations:** 1 Faculty of Health Sciences, Universidad de León, 24071 Leon, Spain; 2 Department of Health Sciences, European University Miguel de Cervantes, 47012 Valladolid, Spain; 3 Research Center on Physical Disability, ASPAYM Castilla y León, 47008 Valladolid, Spain; 4 Institute of Biomedicine (BIOMED), Universidad de León, 24071 Leon, Spain; 5 Physiology Department, University of the Basque Country, 48940 Leioa, Spain

**Keywords:** multiple sclerosis, walking ability, physical training, fatigue, 6MWT

## Abstract

Physical capacity provides a link between disease or impairment and limitations in activity; in multiple sclerosis (MS), it is limited and decreased. The aim of this study was to study the effects of exercise and transcranial direct current stimulation (tDCS) on the left dorsolateral prefrontal cortex area in MS patients with fatigue and an impaired gait ability. A cross-over design was carried out on fifteen patients with two disability associations, but three were excluded. Before and after each intervention, the 6 min walk test (6MWT) and the 2 min walk test (2MWT) were used to assess walking ability and the Modified Fatigue Impact Scale (MFIS) was used to assess fatigue. A total of twelve patients were enrolled (48.0 median age, Kurtzke Disability Scale (EDSS) 3.66 ± 1.3): five females and seven males. After the application of the exercise program, significant improvements were observed in the 6MWT (*p* < 0.001, g = 0.159) and 2MWT (*p* < 0.001, g = 0.182). Furthermore, fatigue was significantly reduced after the application of the exercise program (*p* < 0.05, g = 0.742) and after tDCS (*p* < 0.05, g = 0.525). We could consider therapeutic exercise in the future to improve the walking ability and fatigue in MS patients. Furthermore, tDCS did not exert a significant improvement in walking ability, but it appeared to influence fatigue. Clinical trial registration code: ACTRN12622000264785.

## 1. Introduction

Physical capacity provides a link between disease or impairment and activity limitations [1]. Likewise, if we focus on the individual, physical capacity is a means by which they maintain their ability to carry out their functions. In multiple sclerosis (MS), physical capacity is limited and decreased, which results in various limitations, most notably the impact on the ability to walk. In MS, gait disturbances are so common that after 10–15 years of disease development, 80% of patients have difficulty walking [2,3,4]. Another common symptom in this population is fatigue, which we understand as a subjective lack of physical and/or mental energy. The European prevalence of this variable in MS is 83 per 100,000, and 55% of sufferers say it is the worst symptom they have experienced. This is the reason why these disturbances cause a decrease in the general state of health, which, together with the social and psychosocial conditioning factors, results in a lower quality of life [2,5,6].

From this perspective, one of the most commonly used measures of physical capacity is the ability to walk. Thus, one of the therapies that improves the ability to walk is physical exercise, because exercise can modify the anti-inflammatory effect of MS and may even slow down its progression. For this reason and for its ability to reverse the number of relapses, physical exercise is recommended in this population [5,7,8,9,10]. Moreover, Molt et al. [11] established a bidirectional relationship between physical activity, symptomatology, functional limitations and disability, finding that subjects with mild symptoms had higher levels of physical activity. Current evidence suggests that physical rehabilitation based on exercise could improve physical functions and the walking ability, probably by up to 6 points on the Kurtzke Disability Scale (EDSS) [12,13].

In this sense, another therapy with encouraging results to treat motor disorders and their performance is transcranial direct current stimulation (tDCS). A recent review suggests considering this therapy to train the walking ability [14]. Moreover, an innovation brought by this therapy is the ability to generate long-term potentiation phenomena, causing specific changes in the synaptic efficacy of the targeted brain region [15]. The effect of tDCS on gait improvement has shown positive results, in which anodal-type stimulation leads to the improvement of motor impairments, although all of them have been evaluated over the primary motor cortex (M1) [16,17,18,19]. In contrast, other non-invasive brain stimulation techniques, such as transcranial magnetic stimulation, have targeted stimulation to the left dorsolateral prefrontal cortex (DLPFC), obtaining significant changes in gait parameters in MS patients [20].

In view of this, and following the treatment model used in other studies, this study focuses on the effects of tDCS in the DLPFC area and a concurrent training program for the improvement of gait ability and fatigue [21,22]. The relationships between walking ability, fatigue and sociodemographic data will also be studied. Our hypothesis is that the application of exercise could be more effective than the application of tDCS in terms of improving the walking ability, which could have a more significant impact on their physical capacity. This is because there are currently many studies that claim that exercise is beneficial for improving gait; however, no study has evaluated its effectiveness on this variable through the application of tDCS in the DLPFC. Therefore, we believe that this evaluation could be interesting and at the same time could be compared with the application of exercise.

## 2. Material and Methods

### 2.1. Design and Participants

This is a pilot crossover design of tDCS therapy compared to an exercise program for gait and fatigue performance. Ethical approval was obtained from the University of León (ULE 010-2020). The study was carried out in accordance with the Ethical Principles for Medical Research Involving Human Subjects outlined in the Declaration of Helsinki. All participants were fully informed about all experimental procedures and signed the written informed consent form prior to participation. This clinical trial is registered in a WHO-approved public trials registry, the Australian New Zealand Clinical Trials Registry (ANZCTR), registration number: ACTRN12622000264785.

The study was aimed at all participants with a diagnosis of MS, with no type of MS being excluded. Eligibility criteria included being aged 18 years or older, the presence of fatigue as assessed by the Modified Fatigue Impact Scale (MFIS) [23] (score of 38 or more) and the ability to independently walk (with or without an assistive device) for at least 20 m. Potential participants were excluded if they had any muscular disease or respiratory or cardiovascular risk that might affect the exercise program [24]. Furthermore, the sample selection and subsequent application of the interventions were carried out at the Palencia headquarters of Aspaym Castilla y León (Spain) and at the multiple sclerosis association of Palencia (Spain).

Thus, the first therapy was applied in March 2020, but the COVID-19 pandemic forced the suspension of the treatment. Therefore, the application of tDCS was resumed on 8 June 2020 and its application ended on 28 August 2020. After a washout period of 5 months, the second therapy was applied, which consisted of an exercise programme following the guidelines of Muñoz et al. [21,22], and data collection was ended on 19 April 2020.

### 2.2. Interventions

After the enrolment, an information sheet about the study was distributed and a socio-demographic questionnaire was filled in.

**Figure 1:**
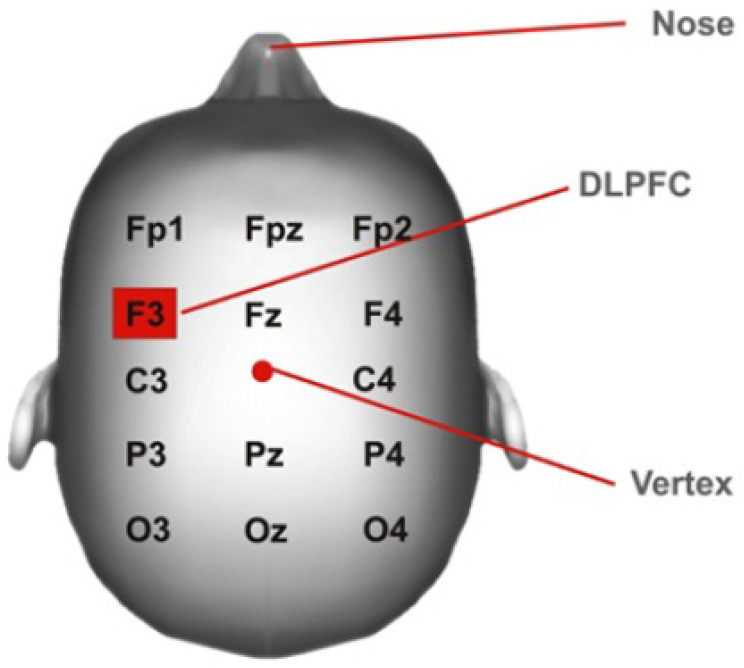
DLPFC (dorsolateral prefrontal cortex) position.

In more detail, the tDCS protocol was applied by a specialised physiotherapist during 10 sessions lasting 20 min, distributed over 4 weeks. The tDCS was delivered with an HDCstim stimulator (Newronika, Milan, Italy) connected to a pair of saline-soaked 35 cm^2^ sponge electrodes placed on the scalp. Current application points were chosen following the 10–20 EEG system, using the protocol described by DaSilva [25] (Figure 1). According to this system, the cathode was located in the right supraorbital cortex, whereas the anode was located in the region corresponding to F3, the left DLPFC region.

The procedure was divided into 4 time points, with T0 corresponding to the baseline, where the 6MWT, 2MWT, IPAQ-SF, EDSS, MFIS and a questionnaire for the collection of clinical, anthropometric and sociodemographic data were administered. After this period, all participants were stimulated with the tDCS by a neurology-specialised physiotherapist and moved to T1, where 6MWT, 2MWT and MFIS were re-evaluated. Following the first intervention, a washout period was applied, which is necessary to prevent the effects of the first intervention from interfering with the second. In our case, we selected a period of 5 months, as benefits of up to 3 weeks have been observed after the application of tDCS. On the other hand, it has been shown that the effect of tDCS is accumulative and that this effect is important to produce the necessary adaptations, so a long washout period was chosen to avoid these effects interfering with those of the other therapy. This was followed by T2, corresponding to the assessment of 6MWT, 2MWT and MFIS, before the exercise programme intervention. Finally, the exercise programme was applied by a neurology-specialised physiotherapist, followed by initiation of the last period, T3, where 6MWT, 2MWT and MFIS were evaluated for the last time.

In summary, the timepoints are T0 = Baseline/Pre-tDCS; T1 = Post-tDCS; (washout); T2 = Pre-Exercise; and T3 = Post-Exercise.

Following this period, the walking ability and fatigue data were collected once more, and the exercise therapy was applied by a neurology-specialised physiotherapist for a period of 4 weeks. Table 1 describes the exercise program protocol, which was divided into strength and endurance.

The training programme consisted of strength work and endurance work. Each subject started the first week with two sessions of strength work on alternate days and one session of resistance work. As we can see, the sessions were increased in both training sessions up to three sessions per week in the fourth week of training. As for the strength training characteristics, the subjects began by performing two circuits composed of the same six exercises, with 15 repetitions of each exercise and 2 min of rest between exercises. Meanwhile, in weeks 3 and 4, they performed three circuits with the same exercises, with 10 repetitions of each exercise and 3 min of rest between exercises. The exercises in each circuit consisted of pushing and pulling exercises, trunk and hip exercises and upper- and lower-limb exercises. In this way, two circuits were developed; these required the work of the same muscle groups but in different starting positions, which facilitated the implementation of the exercises for those participants with some functional limitations. Resistance training was increased from 10 min in week 1 to 30 min in week 4. During weeks 3 and 4, there was a 5 min break in the middle of the session. In addition, the intensity used was moderate, which corresponds to a level of 3–5 on the rate of perceived exertion (RPE) on the Borg scale [26]. The equipment used was a static bike or MOTOmed^®^ kinesiotherapy equipment (RECK-Technik GmbH and Co., Betzenweiler, Germany) depending on the participant’s preference. Finally, walking ability was assessed and fatigue data were collected.

### 2.3. Outcome Measures

The 6MWT and 2MWT were administered following the established instructions for people with MS. The subjects were instructed to walk as fast and as far as possible for 6 min over a distance of 30 m marked by plastic cones. The investigator followed the subject with a measuring wheel to subsequently record the distance travelled, which was marked at 2 min and 6 min. According to the protocol developed for MS, the participants were not encouraged during the test [27,28].

The International Physical Activity Questionnaire Short Form (IPAQ-SF) was used to assess physical activity. This questionnaire has seven items, where six of the items measured the frequency and duration of vigorous, moderate and walking activities during the last 7 days. The 7th item on the IPAQ-SF measured the duration (minutes each day) of time spent sitting on a typical weekday. Its reliability and validity have been investigated in different countries and with different types of populations, including in MS subjects [29,30].

The Kurtzke Disability Scale (EDSS) is considered a well-defined scale. The total score in EDSS is based on an interview performed by the clinician and a neurological examination. The EDSS consists of 20 steps with increments of 0.5: “0” indicates normal neurological examination, while “10” indicates death due to MS [31,32].

The Spanish version of the Modified Fatigue Impact Scale is known as the the MFIS. This scale is a 21 item scale with 9 “physical” items, 10 “cognitive” items and 2 “psychosocial” items. This scale is recommended in the American Multiple Sclerosis Council for Clinical Practice guidelines [23,33].

### 2.4. Statistical Analysis

A sample size calculation was carried out by calculating the difference between dependent groups utilizing G*Power-3.1.9.2 software (G*Power©, Dusseldorf University, Düsseldorf, Germany) and with an α-error of 0.05 and a B error of 0.20. These data produced a sample of 27 subjects. Nevertheless, after the COVID-19 pandemic, many of the participants with MS stopped attending their usual rehabilitation centres, which made recruitment difficult. For this reason, it was decided to use the crossover design. Furthermore, the results found in these patients should be considered primary and the study should be considered a pilot study.

The collected data were analysed using the statistical package IBM SPSS version 24 (SPSS 24, SPSS Inc., Chicago, IL USA). Descriptive analyses were generated for the demographic and clinical variables, the data for continuous variables presented as ± standard deviation (SD) and categorical variables as frequencies (percentages). The normal distribution of the continuous variables was assessed using the Shapiro–Wilk test. The Wilcoxon signed-rank test was used to analyse the results obtained after applying tDCS and the exercise program in the 6MWT, 2MWT and MFIS tests, and Spearman’s correlation (r) was used to determine correlations between the variables (disability, physical activity, fatigue and walking capacity) and the rest of the descriptive variables, with r values showing high (±0.80), moderate (±0.50) and weak (±0.20) differences [34].

The effect size was calculated to express the magnitude of differences between samples, expressed as Hedges’ g (scale: 0–1). The effect sizes were set as small (0.2–0.5), medium (0.5–0.8) and large (>0.8) [34].

The significance level for all tests was set at *p* < 0.05.

## 3. Results

**Figure 2:**
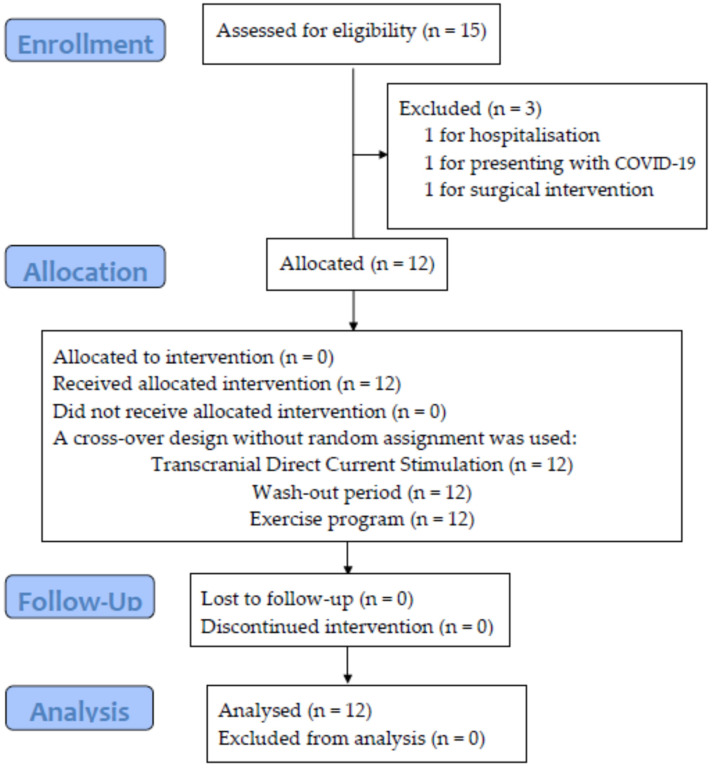
Flow diagram.

Out of the fifteen patients assessed for eligibility, three were excluded (one was hospitalised, one had COVID-19 and one underwent surgery), preventing them from carrying out the exercise program. Finally, twelve patients provided informed consent and enrolled in the study. The types of MS were relapsing–remitting or secondary progressive, patients’ ages ranged from 35 to 66 years and EDSS levels ranged from 0.5 to 5. A flow chart of the enrolment and randomisation process, according to the CONSORT guidelines, is presented in Figure 2. Additionally, Table 2 shows the initial characteristics of the participants.

The results of this study showed significant improvements after the application of exercise in the 6 min walk (*p* = 0.004, g = 0.159) and 2 min walk (*p* = 0.002, g = 0.182) scales, although the effect size was small (Table 3). It should be noted that changes in the 6MWT distance in excess of 30.5 m can be considered clinically significant, indicating that there has been a real change in the 6MWT distance achieved. For the 2MWT scale, a change of 12% or more indicates a clinically significant change. On the other hand, we will take into account the minimally important difference (MID), which refers to the smallest change in score that is perceived as important. With respect to the MID, it has been established for the 6MWT scale that a variability of 19.7 m shows improvement, while a reduction of 7.2 m indicates deterioration.

**Figure 3:**
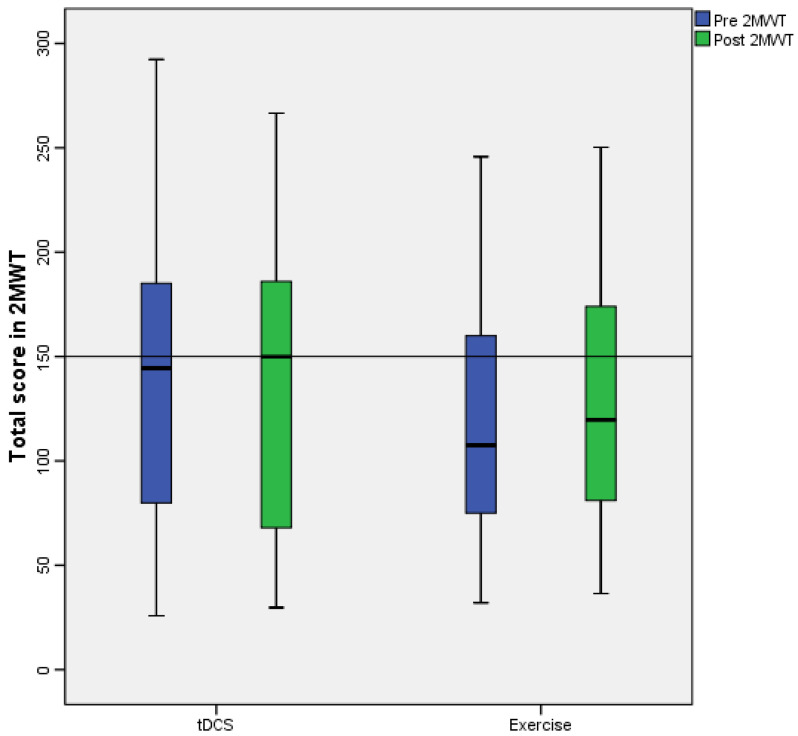
Box and whisker plot of the scores obtained by the participants in the 2MWT.

The median score obtained by participants in the 2MWT tended to improve after the implementation of tDCS and the exercise program. The measurement prior to the application of tDCS was 144.45 (IQR: 69.92–188.67), and after the application of tDCS, it was 149.95 (IQR: 65.25–187.85). The measurement prior to the application of the exercise program was 107.45 (IQR: 70–163.5), and after the application of exercise, it was 119.70 (IQR: 76.2–175.8) (Figure 3).

**Figure 4:**
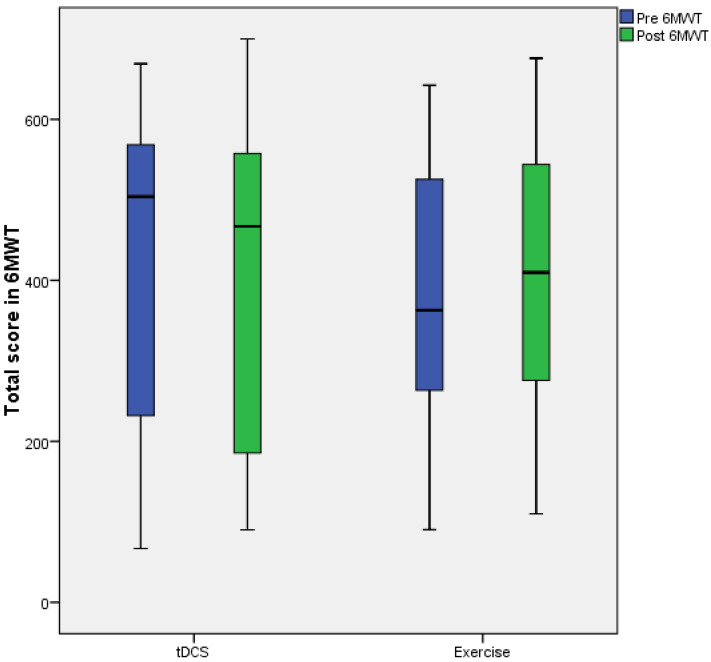
Box and whisker plot of the scores obtained by the participants in the 6MWT.

However, the median score obtained by participants in the 6MWT only tended to improve after the implementation of the exercise program. Thus, the measurement prior to the application of tDCS was 504 (IQR: 198–577.5), and after the application of tDCS, it was 467.1 (IQR: 182.47–565.9). Meanwhile, the measurement prior to the application of the exercise program was 362.8 (IQR: 251.82–538.05), and after the application of exercise, it was 409.65 (IQR: 257.22–547.57) (Figure 4).

It is important to note that after tDCS, the data indicated impairment in three subjects in the 6MWT scales. In addition, three subjects also scored worse on the 2MWT scale. In the meantime, in the 6MWT scale after tDCS and the exercise program, some subjects obtained an MID that indicated a trend towards improvement, without reaching the values to consider it clinically significant. Similarly, we must consider that the mean of the group evaluated after tDCS was higher on the 2MWT scale, so there were clinical improvements, although they were not clinically significant.

A secondary analysis showed significant reductions in fatigue after the application of the exercise program (*p* = 0.03, g = 0.742) and tDCS (*p* = 0.028, g = 0.525). On the other hand, there were no correlations between the sociodemographic variables, IPAQ, 6MWT and 2MWT. The only correlation found was between 6MWT and 2MWT (r = 0.923, *p* < 0.001) and between MFIS and 6MWT (r = 0.605, *p* = 0.037).

## 4. Discussion

In view of the possible interest in the results and their relevance, we present the results of our pilot study as preliminary results. In this way, our principal objective was to study the effects of tDCS on the DLPFC area and the exercise program on the improvement of the gait ability. In this sense, significant improvements were observed in the 6MWT test and 2MWT after the exercise program.

To the best of our knowledge, at the time of writing, the present study is the first to evaluate walking ability by separately applying a concurrent training program and tDCS on the left DLPFC area.

Gait impairment in MS is mainly expressed as a reduction in walking speed, an increased variability in hip, knee and ankle kinematics, a decreased endurance and an impaired postural control. All of this leads to an increased metabolic cost of walking. When we add the variable fatigue, present in the majority of these subjects, we encounter two of the main limitations that are also correlated [27]. Therefore, we have applied therapies that have reported beneficial effects on both fatigue and gait. Nevertheless, our results indicate that the effects of these two variables were small, so we cannot affirm that the training program used as a therapy is clinically relevant. However, there were changes in these scales that are important to take into account at the clinical level. For the 6MWT scale, it has been established that changes in distance exceeding 30.5 m can be considered as clinically significant. On the other hand, the minimum important difference (MID) for people with MS is established as a distance greater than 19.7 m for improvement and a distance lower than 7.2 m for deterioration [35,36]. Our results showed that nine of the subjects exceeded the MID, and five of them exhibited a change greater than 30.5 m, which suggests a clinically significant change. It is true that one of the subjects obtained a lower distance than at the beginning of the treatment, but this did not reach the level of deterioration, since it was not more than 7.2 m. For the 2MWT scale in MS, it is determined that a change of 12% or more indicates a clinically significant difference [37]; our results indicated that all subjects obtained improvements in this scale, although only three of them showed a change of more than 12%.

Moreover, it is known that the 6MWT distance is associated with measures of gait performance and measures of physical fitness [38]. Therefore, our results indicate that although the exercise program is not clinically relevant, it conveys a significant improvement by working on aerobic and muscular skills. In this way, our study, in line with other authors such as Goldman [27], found a correlation between 6MWT and fatigue. The difference is that Goldman’s study also found a correlation between the 6MWT scale and the physical fatigue subscale of the MFIS in MS. However, our results did not show this correlation, although they do reflect an improvement in both walking capacity and fatigue. This may indicate the need to include a larger number of subjects in future studies.

On the other hand, several studies affirm that the application of an exercise program can be beneficial in MS, and, more specifically, that the application of combined training such as the one used by us has the ability to improve the walking capacity in MS [2,10]. For this reason, in the strength part, it is also important to stimulate secondary and stabilising muscles of the hip, trunk and upper limbs to work on coordination and stability in the sagittal and frontal planes [39]. Despite having applied this, we did not obtain clinically significant changes in all the subjects; this may be due to the association between speed and gait pattern and because those subjects with a poor gait prior to training need a longer training duration [40,41]. In this way, several studies use a training period of fewer than 8 weeks [42,43,44], which is effective as long as the volume of work increases from three sessions per week. Our study uses this approach, although only two sessions were performed in the first week, which could be another reason why some of the subjects did not obtain significant improvements.

No significant improvements were obtained after tDCS application, and three of the subjects displayed deterioration in both gait scales. In addition, two of them also exhibited a worsening in the fatigue scale after the application of tDCS, but not after the training program. This may be due to the inter-subject variability that exists in non-invasive brain stimulation techniques and to the so-called “non-responders” who do not respond to the expected corticospinal excitability after the application of the stimulation [45]. Nevertheless, no other study has evaluated the effectiveness of tDCS in the left DLPFC area on walking ability, although our results suggest that this stimulation is not effective. In this way, another area in which we should discourage the use of this stimulation is in the cerebellum, since Nguemaeni’s group [46] found no effect on locomotion, even though they only applied a single stimulation session. Therefore, in future applications, we should follow the recommendations made by other authors who obtained improvements in the 6MWT and 2MWT scales when applying a current to M1 [16,17,18,19]. New trends in this field include the combination of tDCS with aerobic physical activity, resulting in improvements in gait in MS, which may enhance the rehabilitation of this population group [18,47].

It is necessary to consider that the data obtained are preliminary results, so they should be read with caution regarding the significance of the findings, and the study should be considered as a pilot study. This is mainly due to the fact that the study design has a small sample size and no long follow-up over time.

### 4.1. Practical Application

The main strength of this study is the innovation of separately applying a concurrent training program and tDCS on the left DLPFC area to assess walking ability. As for separate therapies, we can highlight that in the strength work, we did not focus only on working the knee muscles, but we also worked secondary and stabilising muscles such as the trunk, hip and upper limbs to achieve greater coordination and stability. On the other hand, no other studies have evaluated the walking ability by applying stimulation to the left DLPFC area. Although our results suggest that this stimulation is not effective, it would be interesting to delimit the areas on which we should focus according to our work objective. This could help the future development of more detailed protocols for the improvement of physical capacity in MS, even though more studies are needed to confirm these data.

### 4.2. Future Line of Applications

In recent years, the development, study and application of different therapies in neurological pathologies such as MS has generated interest. However, despite demonstrating the benefits of therapies such as exercise training, the current recommendations lack specificity. In terms of gait training, as we have already seen, a combined exercise program is a good option, and it is important that the secondary and stabilising muscles are worked. Other therapies such as virtual reality, robot-assisted gait training or tDCS have been used to improve the walking ability. There are some studies that have evaluated the combined application of tDCS with walking or an exercise program in other pathologies, with positive results in stroke patients [16]. This combination of therapies has also been studied in MS with promising results, suggesting that there could be a prolonged beneficial effect induced by the combination of these rehabilitation therapies [47]. Therefore, a future line of research in MS would be to further study the combination of these therapies to determine whether tDCS can improve the efficacy of treatment. Another difficulty we encounter is the disability of the subjects, so another challenge is to adapt the treatments and clinical recommendations to the different degrees of disability [10,48,49,50].

In conclusion, although there are serious limitations due to the small sample size and a lack of long-term assessments, these procedures could be considered as a future strategy to improve walking ability and fatigue in MS patients. In this way, a combined exercise program could improve walking ability and fatigue in patients with MS. Furthermore, tDCS did not show significant improvements in walking ability, but it could improve fatigue.

## Figures and Tables

**Table 1:** Exercise program.

	Week 1	Week 2	Week 3	Week 4
Endurance	1 Ss	2 Ss	2 Ss	2–3 Ss
	10 min	15 min	20 min (5 min of rest in the middle of the session)	30 min (5 min of rest in the middle of the session)
	RPE 3–5	RPE 3–5	RPE 3–5	RPE 3–5
	Static bike/MOTOmed	Static bike/MOTOmed	Static bike/MOTOmed	Static bike/MOTOmed
Strength	2 Ss	2 Ss	3 Ss	3 Ss
	15 Rep (rest 2 min)	15 Rep (rest 2 min)	10 Rep (rest 3 min)	10 Rep (rest 3 min)
	2 Circuits (rest 3 min)	2 Circuits (rest 3 min)	3 Circuits (rest 5 min)	3 Circuits (rest 5 min)

Development of exercise program. Ss = session. RPE = rate of perceived exertion. min = minutes. MOTOmed = aerobic kinesiotherapy equipment. Rep = repetition.

**Table 2:** Clinical and sociodemographic characteristics of participants.

Variables	N [Min-Max]; Mean ± (SD)	Frequency (%)
Age	12 [35–66]; 48.0 ± (8.5)	
Diagnosis (years)	12 [0.8–28]; 16.65 ± (7.44)	
Outbreaks (years)	11 [0–2]; 0.36 ± (0.67)	
Kurtzke Disability Scale EDSS	12 [0.5–5]; 3.66 ± (1.30)	
Walking time (minutes)	9 [0.0–120]; 51.11 ± (41.06)	
Sitting time (minutes)	9 [0–960]; 466.667 ± (304.13)	
6MWT	12 [66.9–669]; 411 ± (207.67)	
2MWT	12 [25.8–292.3]; 139.27 ± (80.98)	
MFIS	12 [38–69]; 44.5 ± (9.69)	
	IPAQ Level	
High level		4 (33.3%)
Moderate level		5 (41.7%)
Low or inactive level		3 (25%)
Type of sclerosis		
Relapsing–Remitting		7 (58.3%)
Progressive Secondary		5 (41.7%)
Outbreak intensity		
Mild		2 (18.2%)
Moderate		1 (9.1%)
Intense		1 (9.1%)
No outbreaks		7 (63.6%)
Medical recommendation		
Physical activity		6 (11.3%)
Other		1 (8.3%)
No recommendation		5 (41.7%)
Fatigue medication		
Yes		5 (41.7%)
No		7 (58.3%)
Rehabilitation		
Yes		11 (91.7%)
No		1 (8.3%)
Intensity rehabilitation		
Occasional		5 (41.7%)
Periodic		7 (58.3%)
Exercise habits		
Occasional		2 (16.7%)
Periodically		10 (83.3%)

Clinical and sociodemographic characteristics of participants. N: number of subjects; SD: standard deviation; 6MWT: six-minute walk test; 2MWT: two-minute walk test; MFIS: Modified Fatigue Impact Scale.

**Table 3:** Pre–post tDCS and pre–post exercise paired samples test for gait capacity and fatigue.

	T0 Median [Range]	T1 Median [Range]	*p*	Size Effect Hedges’ g	T2 Median [Range]	T3 Median [Range]	*p*	Size Effect Hedges’ g
6MWT	504 [602.1]	467.1 [610]	0.388	0.632	362.8 [552]	409.65 [566]	0.004 **	0.418
2MWT	144.45 [266.5]	149.95 [201.35]	0.347	0.027	107.45 [175.7]	119.7 [213.8]	0.002 **	0.182
MFIS	39.5 [31]	38.5 [45]	0.028 *	0.525	43 [33]	36 [52]	0.003 **	0.742

Non-parameter statistic. Wilcoxon signed-rank test. 6MWT: six-minute walk test. 2MWT: two-minute walk test. MFIS: Modified Fatigue Impact Scale. T0: treatment before transcranial direct current stimulation. T1: treatment after transcranial direct current stimulation (tDCS). T2: treatment before exercise. T3: treatment after exercise. * *p* < 0.05 and ** *p* < 0.01.

## Data Availability

The datasets generated during and/or analysed during the current study are available from the corresponding author upon reasonable request.

## Abbreviations

MS	Multiple sclerosis
tDCS	Transcranial direct current stimulation
M1	Primary motor cortex
DLPFC	Left dorsolateral prefrontal cortex
MFIS	Modified fatigue impact scale
6MWT	Six-minute walk test
2MWT	Two-minute walk test
IPAQ-SF	International physical activity questionnaire short form
EDSS	Expanded disability status scale
MID	Minimally important difference
